# The Impact of *Punica granatum* Linn and Its Derivatives on Oxidative Stress, Inflammation, and Endothelial Function in Diabetes Mellitus: Evidence from Preclinical and Clinical Studies

**DOI:** 10.3390/antiox12081566

**Published:** 2023-08-04

**Authors:** Kabelo Mokgalaboni, Sanele Dlamini, Wendy N. Phoswa, Perpetua Modjadji, Sogolo L. Lebelo

**Affiliations:** 1 Department of Life and Consumer Sciences, College of Agriculture and Environmental Sciences, University of South Africa, Florida Campus, Roodepoort 1709, South Africa; 2 School of Chemicals and Physical Sciences, Faculty of Agriculture and Natural Science, University of Mpumalanga, Mbombela 1200, South Africa; 3 Non-Communicable Diseases Research Unit, South African Medical Research Council, Tygerberg, Cape Town 7505, South Africa

**Keywords:** *pomegranate*, diabetes mellitus, oxidative stress, inflammation, endothelial function

## Abstract

Diabetes mellitus is recognized as the leading contributor to cardiovascular disease and associated mortality rates worldwide. Despite the use of pharmaceutical drugs to treat diabetes, its prevalence continues to rise alarmingly. Therefore, exploring remedies with a lower toxicity profile is crucial while remaining safe and effective in addressing this global public health crisis. *Punica granatum Linn* (*pomegranate*), known for its properties and safety profile, has been investigated in applied research and preclinical and clinical trials. However, conflicting reports still exist regarding its effects in diabetes. According to our knowledge, no systematic review has been conducted to critically analyze evidence from preclinical and clinical trials simultaneously, explicitly focusing on oxidative stress, inflammation, and endothelial function in diabetes. Therefore, in this systematic review, we searched for evidence on the impact of *pomegranate* in diabetes using databases such as PubMed, Scopus, and Google Scholar. Our inclusion criteria were limited to studies published in English. Of the 170 retrieved studies, 46 were deemed relevant and underwent critical analysis. The analyzed evidence suggests that *pomegranate* has the potential to alleviate oxidative stress, inflammation, and endothelial dysfunction in diabetes. Although a beneficial impact was noted in these markers, the endothelial function evidence still requires validation through further clinical trials with a powered sample size.

## 1. Introduction

**Figure 1:**
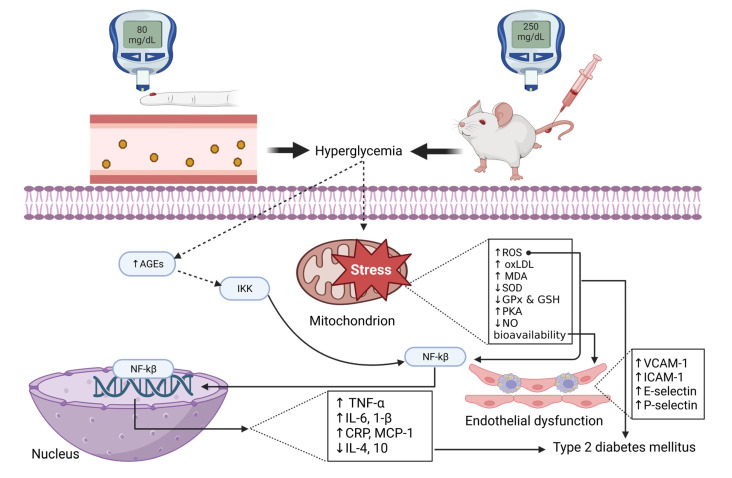
An overview of the pathophysiology of endothelial dysfunction and type 2 diabetes. AGEs: advanced glycation end product; IKK: inhibitor of nuclear factor-κ; NF-κβ: nuclear factor kappa β; ROS: reactive oxygen species; oxLDL: oxidized lipoprotein; MDA: malondialdehydes; SOD: superoxide dismutase; GPx: glutathione peroxidase; GSH: glutathione; PKA: protein kinase; NO: nitric oxide; TNF-α: tumor necrosis factor-alpha; IL: interleukin; CRP: C-reactive protein; MCP-1: monocyte chemoattractant protein-1, VCAM-1: vascular adhesion molecules-1; ICAM-1: intercellular adhesion molecule-1.

Diabetes mellitus (DM) is a chronic metabolic condition characterized by hyperglycemia, which causes damage to the heart, blood vessels, eyes, kidneys, and nerves over time [1]. A report by the International Diabetes Federation (IDF) indicated that about 537 million people were living with DM in 2021, which is anticipated to reach 783 million by 2045 [2]. The IDF further revealed that about 90% of the DM population has type 2 diabetes globally (T2D) [2]. Some of the mechanisms implicated in the pathophysiology of T2D include oxidative stress, which disrupts insulin signaling, damages pancreatic β-cells, and induces inflammation, thus promoting endothelial dysfunction [3,4]. In oxidative stress, there is an imbalance between the rate of reactive oxygen species (ROS) production and the body’s ability to eliminate them [5]. This further predisposes the body to damage due to excessive ROS. Notably, endothelial dysfunction in T2D increases the risk of developing secondary complications and cardiovascular disease (CVD) (Figure 1). 

The literature suggests that any therapeutic approach that can ameliorate oxidative stress may help control T2D and associated CVD. Several pharmaceutical drugs, including sodium-glucose cotransporter-2 (SGLT2) inhibitors, biguanides, glitazones, and α-glucosidase inhibitors, are widely used to control insulin sensitivity and reduce blood glucose in T2D patients [6,7]. However, such drugs are associated with various adverse effects and related complications, ranging from megaloblastic anemia and neuropathy associated with vitamin B_12_ deficiency, increased low-density lipoproteins (LDL), hypoglycemia, water retention, acidosis, bone fractures, heart failure, weight gain, and gastrointestinal reactions [6,7,8,9]. Due to all these secondary complications associated with pharmacological drugs, it would be ideal to evaluate the effect of functional foods and natural compounds with antioxidants on diabetes control and management. In fact, the World Health Organization (WHO) acknowledges the benefits of traditional, complementary, and alternative medicines (TCAMs), especially the use of plants that have been scientifically proven to be effective [10]. This has prompted more research into natural remedies for diabetes.

In South Africa, traditional medicine has existed for a long time, with practitioners providing care to the public, although some of these medicinal plants are not properly verified by botanists for use in diabetes [11]. More recently, Mokgalaboni et al. [12] reported a significant effect of *Corchorus olitorius* in animal models of diabetes on hyperglycemia, oxidative stress, and inflammation. Although the results showed potential benefits in diabetes, this was only conducted in preclinical models, thus calling for more research in clinical trials. Although there are limitations associated with the translation of preclinical studies into clinical trials, the evidence from these studies may provide the basis for developing new alternative therapies to treat DM and prevent secondary complications. *Pomegranate* is another fruit that has gained research attention due to its antioxidant properties and, more recently, has been suggested to be a functional food due to its multiple health-promoting properties [13,14]. This fruit is scientifically known as *Punica granatum Linn* and belongs to the Plantae kingdom and Punicaceae family [15]. Several studies have investigated the effects of *pomegranate* on oxidative stress in diabetes. The evidence from preclinical studies has demonstrated the potential effect of *pomegranate* as an anti-oxidative agent, as revealed by its ability to reduce the levels of ROS in rodent models of diabetes [16,17,18,19,20,21,22,23,24].

Interestingly, the same results seem to be observable in clinical studies, as revealed by reduced markers of oxidative stress in T2D patients following treatment with *pomegranate* [25,26,27,28,29,30]. However, there are still some inconsistencies in clinical trials on the impact of *pomegranate*, especially on different markers of oxidative stress and inflammation, and limited evidence on endothelial function in diabetes. More recently, a meta-analysis was conducted on *pomegranate*, and the researchers found no effect of *pomegranate* on oxidative stress and inflammation. One of the limitations is that their analysis focused on only total antioxidant capacity (TAC) and high-sensitive C-reactive protein (hs-CRP) as markers of oxidative stress and inflammation, respectively; hence, they might not independently be ideal predictors [27]. In contrast, another meta-analysis showed a significant decrease in hs-CRP, interleukin-6 (IL-6), and tumor necrosis factor-alpha (TNF-α) without effects on CRP, soluble vascular cell adhesion molecule-1 (sVCAM-1), soluble intercellular cell adhesion molecule-1 (sICAM-1), and malonaldehyde (MDA) [31].

Additionally, a meta-analysis by Morvaridzadeh et al. [32] on *pomegranate* showed no effect on TAC, glutathione peroxidase (GPx), paraoxonase-1 (PON1), and MDA. Furthermore, the analyzed studies encompassed a wide range of conditions, making it difficult to interpret and make conclusive remarks and recommendations to a wider diabetic population. The current review highlights the potential benefits of *pomegranate* extracts, focusing on various oxidative, inflammation, and endothelial markers in diabetes while elucidating its mode of action.

## 2. Methodology

### 2.1. Search Strategy

Evidence was retrieved by independent investigators (KM and SD) through online databases, including Scopus and PubMed, on 20 May 2023 and updated on 20 July 2023, according to the updated guidelines of the preferred reporting items for systematic review and meta-analysis (PRISMA) [33]. The exact search strategy is presented in the Supplementary File (Tables S1–S6). The search comprised five key terms: “*pomegranate*”, “oxidative stress”, “inflammation”, “endothelial dysfunction”, and “diabetes mellitus”. This was performed in a separate search with the use of the Boolean operator “AND”. The search was restricted to evidence published in English, however, with no duration limitation. Additionally, two independent investigators verified the search before decisions could be made (WNP and PM).

### 2.2. Study Selection

Two independent researchers (KM and SD) selected the studies based on their title, abstract, keywords, and overall aims and findings. The third independent researcher read all of the studies that the prior researchers had disagreements on and made a conclusion. The studies that passed the initial screening were subjected to a full screening of their full text. All studies that passed the second phase of screening were included if they met the following criteria: (i) they used either *pomegranate* juice extract, powder, or its active compounds; (ii) they included a rodent model of diabetes or diabetic patients; and (iii) they reported any markers of oxidative stress, inflammation, or endothelial function. The exclusion criteria were (i) reviews, commentaries, book chapters, and letters to editors and (ii) in vitro studies.

### 2.3. Data Extraction

Two investigators (KM and SD) independently extracted data from all relevant studies. The data extracted from each clinical study included the first author’s surname; publication year; the country where the study was conducted; the study design; the number of participants in both groups; the number and proportion of males; the mean age in years; the body mass index (BMI) of the group on *pomegranate* in kg/m^2^; the duration of treatment with *pomegranate*; the dose; the form of *pomegranate* treatment; and the main findings. Similarly, for preclinical studies, the data extracted included the first author’s surname, the experimental model, the form of *pomegranate* and the duration of treatment, the main findings, and the statistical data for effect measures. In cases of disparities between the two investigators, WNP made a decision by re-evaluating the study and items in question.

## 3. Results

A total of 95 records were obtained from Scopus, while 57 were retrieved from PubMed. Additionally, Google Scholar was searched, and about 18 records were found relevant. Prior to the screening process, it was determined that 34 records were duplicates found in both databases through these separate searches, and thus, they were excluded. As a result, 136 records were subjected to initial screening by their title, abstract, keywords, and objectives. Fifty-one records were considered irrelevant and excluded due to their title, abstract, and aims being outside the scope of our review.

**Figure 2:**
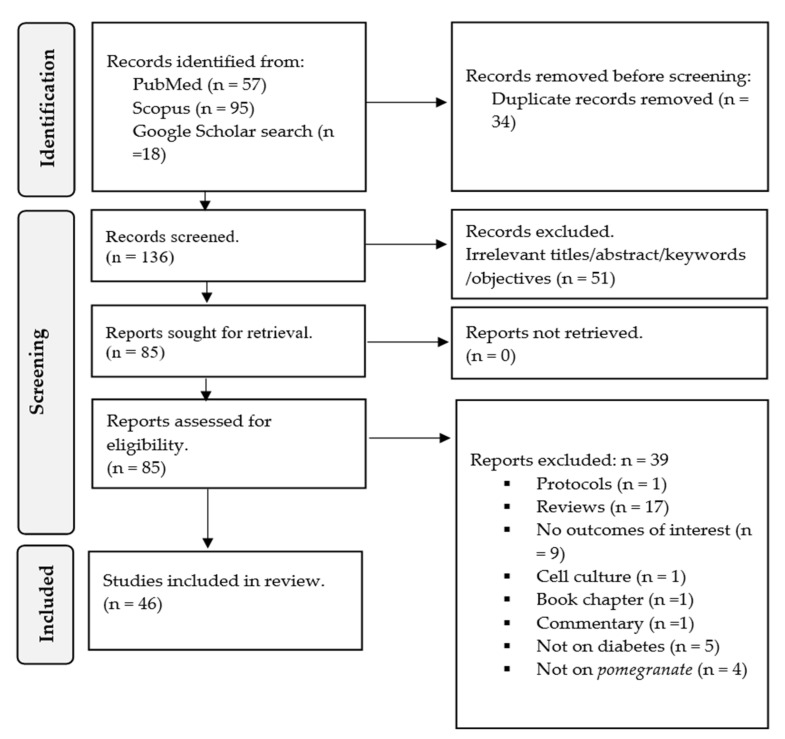
PRISMA flow chart depicting the study selection, screening, and inclusion.

Consequently, a total of 85 records underwent a thorough screening process. Among these 85 records, 39 were excluded based on the following criteria: 17 were review articles, 9 did not report on outcomes of interest, 4 did not focus on *pomegranate* as an intervention, 1 was a commentary, 1 was a book chapter, 5 were not related to diabetes or models, 1 was a cell culture study, and 1 was a study protocol. Hence, a total of 46 records (33 preclinical and 13 clinical trials) were deemed relevant as they reported on the effect measures of interest in diabetic models or patients (Figure 2).

**Characteristics of the included clinical and preclinical studies.**

We identified 33 preclinical studies published in peer-reviewed journals from 2006 to 2023 relevant to our review. These studies were published in various countries, including China [22,34,35], Egypt [36,37,38,39,40,41,42,43,44], India [24,45,46,47,48], Iran [20,23,49,50], Indonesia [51], Israel [52], Iraq [53], Malaysia [16,54], Mexico [55], Saudi Arabia [56], Turkey [19,21,57,58], and United States of America [59]. Various methods were used to induce a diabetes model in these preclinical studies, such as the intraperitoneal injection of single streptozotocin (STZ), STZ combined with nicotinamide (NAD), STZ, and high-fat diet, and alloxan monohydrate. Different rodent models, including rats (Wistar, Sprague Dawley, and Swiss Albino) and mice (C57BL/6, Balb/C, and Kunming), were used in these studies (Table 1).

Additionally, we found thirteen relevant clinical studies that investigated the impact of *pomegranate* on oxidative stress, inflammation, and endothelial function in individuals with diabetes. These studies were published in peer-reviewed journals between 2000 and 2022, thus providing a comprehensive range of evidence on the effects of *pomegranate* in diabetes. The research was conducted in various countries, including three studies in Israel [28,29,60], eight in Iran [25,26,30,61,62,63,64,65], one in Turkey [66], and one in Bosnia and Herzegovina [67]. A total of 468 participants with diabetes, with a mean age of 54.48 ± 1.64, were included in these studies, alongside 120 healthy control individuals. The mean body mass index for the diabetic group receiving *pomegranate* was 29.70 ± 2.42 kg/m^2^, and 145 participants were male. The *pomegranate* was administered orally as juice or a capsule for 6 weeks to 3 months (Table 2). Different study designs were included, with at least four being quasi-experimental; this study aims to evaluate interventions but does not use randomization. One was a case–control study and eight were randomized controlled trials (Table 2).

**An overview of *pomegranate*, its bioactive compounds, and its bioavailability**.

**Figure 3:**
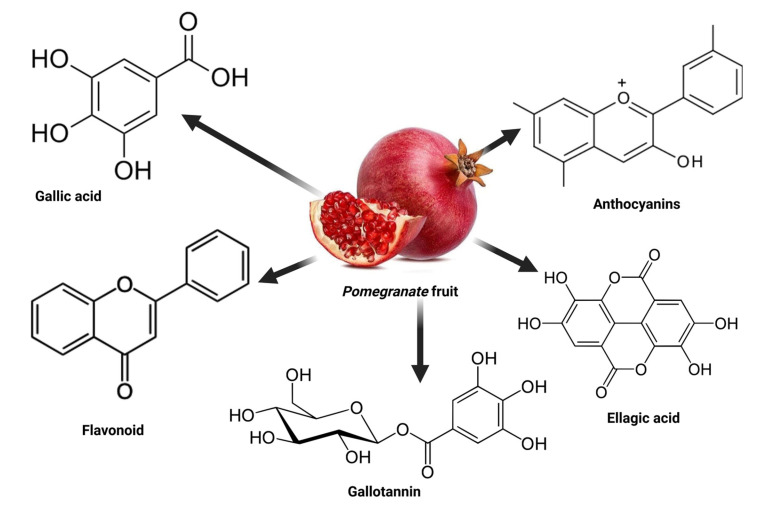
The active compounds found in *pomegranate*.

*Punica granatum* L. (*pomegranate*) is a plant belonging to the Punicaceae family [15]. It is widely cultivated in regions such as the Middle East, the Caucasus, northern and tropical Africa, Iran, the Indian subcontinent, Central Asia, Southeast Asia (in drier parts), and the Mediterranean Basin [68]. Therefore, consuming fruits and vegetables containing these bioactive compounds has been associated with good health [69,70]. Some of these bioactive compounds found in *pomegranate* fruit include hydrolyzable tannins like gallotannins and ellagitannins, along with ellagic acid and its derivatives, gallic acid, anthocyanins, proanthocyanidins, flavonoids, vitamins, sterols, lignans, saccharides, fatty acids, organic acids, terpenes, and terpenoids (Figure 3). The term “bioavailability” refers to the extent to which a chemical or medicine becomes available to its intended target tissue [71]. In the case of *pomegranate*, ellagitannins are metabolized into ellagic acid and glucose in the small intestine [72], while gallotannins are metabolized into gallic acid and glucose [73,74,75]. The predominant hydrolyzable tannin in *pomegranate* is punicalagin [76], in *pomegranate* juice [77,78,79]. The primary phenolic compounds in *pomegranate* seed include flavol-3-ols, flavonoid glycosides, phenolic acids, and hydrolyzable tannins [80,81]. *Pomegranate* peel is abundant in gallic acid and is known for its high phenolic content, and it is also rich in flavonoids, including kaempferol-3-O-glucoside [69]. A fresh *pomegranate* fruit peel contains at least flavonoids (51.52 ± 8.14 mg of rutin), total phenolic compounds (85.60 ± 4.87 mg of gallic acid), anthocyanins (102.2 ± 16.4 mg of cyanidin-3-glucoside), and hydrolyzable tannins (139.63 ± 4.25 mg of tannic acid) [81].

**The effect of *pomegranate* and its derivatives on markers of inflammation in animal models of diabetes.**

Evidence from preclinical studies has revealed, to some extent, the potential of *pomegranate* as an anti-inflammatory remedy. For instance, El-Deeb et al. [41] used Sprague Dawley STZ-NAD-induced diabetic rats to explore the benefits of 200 mg/kg of ethanolic extract of *pomegranate* for four weeks. This study demonstrated a significantly lower level of TNF-α (43.2 ± 1.51 pg/mL) compared to untreated diabetic rats (73.0 ± 0.87 pg/mL), *p* < 0.05. The same researcher further reported significantly decreased IL-6 (46.1 ± 1.31 pg/mL) compared to the untreated group (66.9 ± 0.99 pg/mL), *p* < 0.05. Moreover, these findings were confirmed by a previous study that used the same model [54] with a dose of 1 mL of PJ or 100 mg of *pomegranate* seed powder for 21 days. Interestingly, they reported a significant decrease in markers of inflammation, including TNF-α, IL-6, and NF-κβ. For instance, the mean of TNF-α was 2944.02 pg/mL, *p* > 0.05, and 2844.92 pg/mL, *p* < 0.05, at 1 mL of PJ and 100 mg of PS, respectively, compared to the untreated group, with 3074.37 pg/mL. Additionally, there was a decrease in the levels of IL-6 in the respective dosages: 296.42 pg/mL, *p* < 0.01, and 316.51 pg/mL, *p* < 0.01, compared to 355.86 pg/mL. Furthermore, the same study showed reduced NF-κβ in PJ, 2228.89 pg/mL, *p* < 0.05, and 2138.68 pg/mL in the PS group compared to the untreated group, with 2337.14 pg/mL, *p* < 0.01. Similar results are partly corroborated by Shaker et al. [44], who also used the same model of diabetes and a slightly higher dosage (300 mg/kg) of *pomegranate* seed extract for four weeks. Consistently, they demonstrated a significant decrease in the pancreatic expression of NF-κβ, mean, SD, 765.6 ± 9.9 pg/gm, compared to the untreated group, with 992.5 ± 8.46 pg/gm, *p* < 0.01. Furthermore, transforming growth factor beta (TGF-β) was significantly decreased in the treated group, 477.5 ± 4.24 pg/gm, compared to the untreated group, 879.16 ± 6.91 pg/gm, *p* < 0.01. In another investigation, diabetic Sprague Dawley rats induced by STZ were subjected to an oral *pomegranate* regimen of 150 mg/kg daily for 18 days [56]. In this experiment, the researchers made notable observations, specifically a substantial reduction in embryonic IL-1β, 2.78 ± 0.48 pg/mL, compared to 4.12 ± 0.57 pg/mL and IL-6, 1.30 ± 0.15 pg/mL compared to the untreated group, 2.18 ± 0.29 pg/mL. Moreover, in the diabetic mother rats, the same results were shown. For instance, there was a decrease in IL-6: 3.74 ± 1.08 pg/mL and IL-1β, 5.12 ± 1.24 pg/mL compared to the untreated diabetic groups: IL-6, 6.44 ± 1.98 pg/mL, *p* < 0.05 and IL-1β, 7.99 ± 1.23 pg/mL, *p* < 0.05. These results are further supported by Abo-Saif et al. [43], who demonstrated a significant decrease in IL-1β following eight weeks of PPE at a 150 mg/kg dose in diabetic Wistar rats. El-Missiry et al. [42] used the same model with an intraperitoneal punicalagin dose of 1 mg/kg for 15 days. Consistently, the treatment resulted in a significant decrease in IL-6 (45.9 ± 2.27 pg/mL compared to 72.7 ± 1.98 pg/mL, *p* < 0.001); TNF-α, (37.3 ± 0.42 pg/mL compared to 63.8 ± 2.75 pg/mL, *p* < 0.001); and IL-1β (75.7 ± 2.09 pg/mL compared to 124.9 ± 4.44 pg/mL, *p* < 0.001). In the most recent study by Abdulhadi et al. [53], STZ-induced diabetic Wistar rats were intraperitoneally injected with punicalagin at a dose of 1 mg/kg for 15 days; interestingly, this was associated with a significant decrease in CRP (*p* < 0.001) and MCP-1 (*p* < 0.001) levels compared to the untreated diabetic group. On the contrary, a different study found no significant effect of *pomegranate* oil on CRP (*p* < 0.05) in HFD-fed mice [59].

**The effects of *pomegranate* and its derivatives on markers of endothelial function in rodent models of diabetes.**

Endothelial dysfunction in diabetes increases the risk of developing CVD complications such as atherosclerosis due to the formation of foam cells in the blood vessels [82]. Thus, to curb these secondary complications in diabetes, it is important to control endothelial dysfunction in diabetes. Therefore, *pomegranate* may improve endothelial function due to its antioxidant potential, especially in diabetes. For example, in Sprague Dawley rats with STZ-NAD-induced diabetes, a four-week administration of 200 mg/kg of ethanolic extract of *pomegranate* demonstrated a significant decrease in NO bioavailability: 34.4 ± 0.60 µmol/L, *p* < 0.05 compared to 56.3 ± 1.30 µmol/L [41]. A study by Çukurova et al. [58], also using the same animal model, revealed that 2.5 mL of PJ treatment significantly decreased endothelial nitric oxide synthase (eNOS) expression (*p* < 0.05). Additionally, in Wistar rats with STZ-NAD-induced diabetes, lower levels of NADPH oxidase (NOx) were observed following oral administration of PAJ at 100 (13 ± 1.5 µmol/g) or 300 mg (11.6 ± 1.0 5 µmol/g) compared to 31.7 ± 1.45 µmol/g, *p* < 0.001 [36]. However, Onal et al. [19], after using 100 mg/kg *pomegranate* for ten weeks in Sprague Dawley STZ-induced diabetic rats, observed no significant difference in iNOS (*p* > 0.05) and eNOS (*p* > 0.05) protein levels. Most recently, Abdulhadi et al. [53] investigated 1 mg/kg intraperitoneal injection of punicalagin in STZ-induced diabetic Wistar rats for 15 days. Interestingly, this study reported significantly lower levels of ICAM-1 (*p* < 0.001), VCAM-1 (*p* < 0.001), E-selectin (*p* < 0.001), and endothelin-1 (ET-1) (*p* < 0.001) compared to the untreated diabetic group. The aforementioned results were corroborated by El-Mansi et al. [56], who used the same model coupled with an oral methanolic extract of *pomegranate* at a 150 mg/kg dose for 18 days. This study demonstrated a significant decrease in the level of ET-1 in both the embryo (3.59 ± 0.27 pg/mL) and its mother (6.87 ± 0.41 pg/mL) compared to the untreated diabetic embryo (5.16 ± 0.37 pg/mL) and its mother (8.87 ± 0.68 pg/mL). Increased ET-1 stimulates the production of ROS [83,84]. Its reduction in diabetes following *pomegranate* treatment shows its potential to ameliorate endothelial dysfunction and associated CVD complications.

**The effect of *pomegranate* and its derivatives on markers of oxidative stress in a rodent model of diabetes.**

For instance, in Wistar rats with STZ-induced diabetes, treatment with *pomegranate* seed oil at doses of 0.4 and 0.8 mg/kg for 28 days increased total thiol content and decreased MDA levels in the heart (*p* < 0.01) and kidneys (*p* < 0.001) [23]. Similarly, in an STZ-NAD-induced diabetic model, high doses of *pomegranate* aril juice (100 or 300 mg/kg) administered for six weeks significantly reduced MDA levels (12.03 ± 0.37 nmol/g) at 100 mg/kg and 6.67 ± 0.22 nmol/g at 300 mg/kg compared to untreated diabetic group (13.81 ± 0.44 nmol/g). Consistently, this study reported increased activity of GPx (44.2 ± 7.9 U/g at 100 mg/kg) and (67.1 ± 3.7 U/g, *p* < 0.001 at 300 mg/kg) compared to 26.1 ± 1.7 U/g, *p* < 0.001, glutathione (GSH) (1.54 ± 0.26 mmol/g at 100 mg/kg and 2.34 ± 0.31 mmol/g, *p* < 0.001 at 300 mg/kg versus 1.02 ± 0.06 mmol/g. Concomitantly, the levels of superoxide dismutase (SOD) showed a significant increase at both 100 mg/kg (557.6 ± 10.1 U/g, *p* < 0.001) and 300 mg/kg (611.8 ± 12.4 U/g, *p* < 0.001) compared to the baseline (510.0 ± 6.3 U/g). Similarly, catalase (CAT) levels also exhibited a noticeable increase at 100 mg/kg (1.88 ± 0.03 U/g) and 300 mg/kg (1.83 ± 0.04 U/g) compared to untreated diabetic 1.42 ± 0.12 U/g [36]. Another study using the same model reported that different doses decrease pancreatic thiobarbituric acid reactive substances (TBARS) and MDA levels. For instance, MDA was reduced (0.427 ± 0.034 nmol/mg at 250 mg/kg and 0.325 ± 0.012 nmol/mg at 500 mg/kg) compared to untreated diabetic rats (0.686 ± 0.035 nmol/mg, *p* < 0.001. Interestingly, this was accompanied by increased activities of GPx (*p* < 0.05), GSH (7.31 ± 0.109 nmol/g at 250 mg/kg and 11.36 ± 0.099 nmol/g at 500 mg/kg) compared to 2.68 ± 0.87 nmol/g, CAT (*p* < 0.05), SOD (*p* < 0.05), and glutathione reductase (GR) (*p* < 0.05) [47]. Consistently, in a Sprague Dawley diabetic rat induced by STZ-NAD, treatment with an ethanolic extract of *pomegranate* at a dose of 200 mg/kg for four weeks led to a decrease in MDA levels (14.9 ± 0.43 nmol/mL compared to untreated group, 21.7 ± 0.97 nmol/mL). This was coupled with an increase in markers of antioxidant capacity such as TAC (0.57 ± 0.02 mmol/L compared to 0.29 ± 0.01 mmol/L, *p* < 0.05) and GSH (38.7 ± 0.96 mg/dL compared to 26.0 ± 0.79 mg/dL, *p* < 0.05) [41]. Furthermore, in STZ-induced diabetic Wistar rats, administration of PSO at doses of 0.4 and 0.8 mL/kg for three weeks significantly increased the activity of CAT (*p* < 0.001), SOD (*p* < 0.001), and GPx (*p* < 0.001) in the tissue and mitochondria while decreasing oxidative stress index (OSI) values [49]. These findings are also supported by an experimental study in Wistar diabetic rats that were treated with *pomegranate* peel extract (PSE) at a dose of 300 mg/kg for four weeks, as they also reported a significant increase in pancreatic GSH levels (46.78 ± 1.5 pg/gm compared to 46.78 ± 1.5 pg/gm) [44]. The administration of *pomegranate* as either leaf extracts (100 and 200 mg/kg) increased SOD (2.03 ± 0.28 Umin/mg at 100 mg/kg and 2.47 ± 0.31 Umin/mg at 200 mg/kg) compared to 1.85 ± 0.15 Umin/mg. Likewise, the 100 or 200 mg/kg of fruit peel extract also significantly increased SOD (2.32 ± 0.35 Umin/mg at 100 mg/kg and 2.82 ± 0.29 Umin/mg at 200 mg/kg all compared to 1.85 ± 0.15 Umin/mg) and CAT (60.23 ± 5.26 Umin/mg at 100 mg/kg and 70.23 ± 5.13 Umin/mg at 200 mg/kg both compared to 40.02 ± 4.51 Umin/mg). Furthermore, the same study reported a decrease in TBARS in (2.33 ± 0.62 mmol/g and 1.65 ± 0.28 mmol/g at 100 LEP and PEP, respectively, compared to untreated diabetic rats, with 2.75 ± 0.78 mmol/g, *p* < 0.05) Wistar diabetic rats [48]. According to Tugcu et al. [21], PJ at a dose of 100 μL significantly increased GSH (21.58 ± 1.65 nmol/mg compared to 16.15 ± 2.63 nmol/mg, *p* = 0.010) and GPx (187.95 ± 26.62 nmol/mg compared to 148.09 ± 26.61 nmol/mg, *p* = 0.042) in Sprague Dawley diabetic rats. In addition, the same scholar reported a significant decrease in MDA (0.92 ± 0.09 nmol/mg compared to 1.28 ± 0.11 nmol/mg, *p* < 0.001) without changes in SOD (21.87 ± 3.54 U/mg compared to 20.58 ± 5.34 U/mg, *p* = 0.938). Interestingly, evidence from Wistar diabetic rats treated with PGE at doses of 100, 200, and 350 mg/kg for 21 days revealed a significant reduction in the generation of ROS (*p* < 0.001) [50]. Bagheri et al. [20] also demonstrated a significant increase in GSH (*p* < 0.05), GPx (*p* < 0.05), and SOD (*p* < 0.05), concomitant with decreased CAT (*p* < 0.05) and MDA (*p* < 0.05) when diabetic Wistar rats were treated with ethanolic *pomegranate* extract. Similarly, the recent findings of Abo-Saif et al. [43] showed that the use of *pomegranate* peel extract (PPE) at a dose of 150 mg/kg for eight weeks significantly decreased lipid peroxidation and MDA.

Consistently, El-Missiry et al. [42] showed that intraperitoneal administration of punicalagin at a dose of 1 mg/kg for 15 days in Wistar rats resulted in a significant increase in GSH (*p* < 0.001), CAT (*p* < 0.001), and SOD (*p* < 0.001) activity while decreasing MDA (*p* < 0.001) and hydrogen peroxide levels (*p* < 0.001). Similarly, treatment with *pomegranate* juice or seed extract at a dose of 500 mg/kg for four weeks significantly increased CAT (39.5± 2.27 U/mL, *p* < 0.05, with PJ and 38.20 ± 3.08 U/mL compared to 21.7 ± 2.83 U/mL, *p* < 0.05) activity and decreased MDA (16.37 ± 2.77 µmol/L, *p* < 0.05, and 14.1 ± 2.54 µmol/L, *p* < 0.05, compared to 23.73 ± 3.29 µmol/L, *p* < 0.05) levels in albino rats with alloxan-induced diabetes [37]. Most importantly, three different doses of *pomegranate* hexane extract (25, 50, and 75 mg/kg) given to STZ-induced diabetic Wistar rats for eight weeks significantly decreased MDA levels (142 ± 32 µmol/mg, 133 ± 25 µmol/mg, and 121 ± 25 µmol/mg, respectively, compared to 188 ± 37 µmol/mg, *p* < 0.05). The same study reported increased GSH (259 ± 50 µg /mg, 128 ± 16.4 µg /mg, and 128 ± 16.4 µg /mg, with all values compared to 123.6 ± 19.3 µg/mg, *p* < 0.05). In addition, this study demonstrated increased CAT activity at 25, 50, and 75 mg/kg (843 ± 134 U/mg, 987 ± 56 U/mg, and 1112 ± 86 U/mg, respectively, in comparison with 358± 65 U/mg, *p* < 0.05). Furthermore, these researchers demonstrated a significant increase in SOD according to these dosages (1021 ± 163 U/mg, 836 ± 53 U/mg, and 1233 ± 153 U/mg, respectively, compared to 760 ± 78 U/mg, *p* < 0.05) [38]. When Wistar rats were treated with STZ to induce diabetes and further fed with a ground *pomegranate* flower pellet mixture (300, 400, and 500 mg/kg), decreased levels of lipid peroxidation (LPO) (3.41 ± 0.24, 3.21 ± 0.24, and 2.91 ± 0.38, respectively, compared to 5.54 ± 0.25) were found. The same study reported improved GSH activity (162.9 ± 3.7 µg/g, 172.8 ± 4.0 µg/g, and 179.8 ± 3.3 µg/g in the respective doses compared to 156.1 ± 3.3 µg/g, *p* < 0.05) [57]. Although a study that used 2.5 mL of diluted *pomegranate* juice in STZ-indued diabetic Sprague Dawley rats showed a significant increase in SOD activity (11.28 (9.71–12.85) U/mg) compared to (8.72 (8.07–10)U/mg, *p* < 0.001)), this was associated with no significant changes in GSH (2.28 (1.89–2.69) nmol/mg) compared to the untreated diabetic rats (1.96 (1.64–2.16), *p* > 0.05)) [58]. Another study focused on Wistar rats with STZ-induced diabetes when treated with a *pomegranate* flavonoid fraction at doses of 50 mg/kg, 100 mg/kg, and 200 mg/kg for 28 days [46]. The results showed a significant decrease in MDA levels (289.31 ± 4.70 nmol/mg, 268.55 ± 5.65 nmol/mg, and 229.58 ± 4.03 nmol/mg, all compared to 389.87 ± 6.58 nmol/mg, *p* < 0.01) which was more pronounced at 200 mg/kg. Additionally, the same report showed an increase in GSH (3.97 ± 0.06 U/mg, 4.14 ± 0.04 U/mg, and 4.53 ± 0.06 U/mg, respectively, compared to 2.73 ± 0.06 U/mg, *p* < 0.01) SOD activity [46]. Interestingly, CAT was reportedly increased (42.59 ± 0.74 U/mg, 46.21 ± 0.65 U/mg, and 52.48 ± 0.64 U/mg in the respective dosages compared to the untreated group, 36.64 ± 1.54 U/mg, *p* < 0.001). Lastly, the same study showed an increase in the activities of SOD in all doses as compared to the untreated group (5.03 ± 0.05 U/mg, 5.71 ± 0.13 U/mg, and 6.11 ± 0.12 U/mg, respectively, compared to 3.35 ± 0.16 U/mg, *p* < 0.001).

Similar findings were observed in diabetic rats given oral administration of *pomegranate* leaves ethanolic extract at doses of 50, 100, and 200 mg/kg for 28 days [45]. This led to significantly decreased MDA in all doses (343.38 ± 5.95 nmol/mg, 274.22 ± 6.17 nmol/mg, and 242.69 ± 6.28 nmol/mg, respectively, compared to 448.91 ± 12.12 nmol/mg, *p* < 0.01), with a pronounced reduction observed at 200 mg/kg [45]. Moreover, there was increased activity of GSH (25.92 ± 1.12 U/mg, 32.28 ± 0.92 U/mg, and 36.99 ± 1.09 U/mg, respectively, compared to 13.62 ± 1.07 U/mg, *p* < 0.01), CAT (51.21 ± 0.54 U/mg, 57.52 ± 0.58 U/mg and 66.59 ± 0.57 U/mg, respectively, compared to 43.68 ± 1.41 U/mg, *p* < 0.01), and SOD (11.22 ± 0.69 U/mg, 10.66 ± 0.62 U/mg, and 11.84 ± 0.47 U/mg, respectively, compared to 5.16 ± 0.79 U/mg, *p* < 0.01) [45]. Regarding STZ-induced IDDM in Swiss Albino rats, *pomegranate* at 200 mg/kg for 20 days significantly increased SOD (182.8 ± 11.68 U/mL versus the untreated group 50.06 ± 3.56 U/mL, *p* < 0.001) and TAC (1.40 ± 0.06 mM/L compared to 0.604 ± 0.18 U/mg, *p* < 0.001) [39]. Interestingly, this was accompanied by a reduction in the level of MDA (4.48 ± 1.14 nmol/L compared to 9.84 ± 1.21 nmol/L, *p* < 0.001) [39]. Onal et al. [19] also demonstrated a significant decrease in MDA levels (*p* < 0.05) following the administration of *pomegranate* at a dose of 100 mg/kg for ten weeks in Sprague Dawley STZ-induced diabetic rats. According to El-Mansi et al. [56], the administration of 150 mg/kg *pomegranate* for 18 days to diabetic mothers significantly reduced MDA (*p* < 0.05) and GPx (*p* < 0.05) activity and remarkably increased the activity of CAT (*p* < 0.05) and SOD (*p* < 0.05) enzymes in diabetic Wistar rats. In a study involving STZ-NAD-induced Sprague-Dawley rats, the combination of *pomegranate* juice and seeds (1 mL of PJ + 100 mg of PS) for 21 days significantly increased the levels of enzymatic antioxidants, including CAT, SOD (25.1 ± 0.42 U/mL, 20.8 ± 0.61 U/mL, and 28.9 ± 0.8 U/mL compared to 17.2 ± 0.51 U/mL, *p* < 0.05), and total antioxidant status (TAS) (0.91 ± 0.01 mmol/L, 0.73 ± 0.11 mmol/L, and 1.03 ± 0.03 mmol/L compared to 0.69 ± 0.03 mmol/L, *p* < 0.05), and decreased MDA (*p* < 0.05) in the plasma [16]. Treating STZ-induced diabetic Wistar rats with *pomegranate* juice at doses of 100 mg/kg and 300 mg/kg for four weeks resulted in increased GSH (56.25 ± 1.90 nM/mg and 66.51 ± 1.82 nM/mg compared to 34.67 ± 2.76 nM/mg, *p* < 0.05), CAT (47.14 ± 1.65 U/mg and 66.36 ± 1.99 U/mg compared to 16.47 ± 0.79 U/mg, *p* < 0.05), and SOD levels (165.3 ± 3.25 U/mg, 237.9 ± 19.72 U/mg compared to 45.62 ± 6.10 U/mg, *p* < 0.05) and decreased levels of TBARS (1.247 ± 0.02 μM/mg and 0.80 ± 0.02 μM/mg in respective dosage compared to untreated group 2.23 ± 0.04 µM/mg, *p* < 0.05) [24]. Some contrasting findings were dose-dependent as Praseytastuti et al. [51] revealed that STZ-induced diabetic Sprague Dawley rats for four weeks of *pomegranate* juice only 2 mL/200 g of PJ could significantly decrease MDA levels while 1 and 4 mL/200 g showed no difference in MDA levels (post-treatment, 0.69 ± 0.22 µmol/L compared to baseline 2.02 ± 1.68 µmol/L, *p* > 0.05 at 1 mL/200g). Moreover, at 2 mL/200 g (0.68 ± 0.38 µmol/L compared to 2.02 ± 1.68 µmol/L, *p* < 0.05 at 2 mL/200 g) and 0.85 ± 0.12 µmol/L compared to 3.16 ± 2.09 µmol/L at a dose of 4 mL/200 g. This is partially supported by more recent evidence, as a study by Abdulhadi et al. [53] showed that intraperitoneal injection of punicalagin at 1 mg/kg for 15 days in STZ-induced diabetic Wistar rats showed a significant decrease in MDA formation in the pancreas (*p* < 0.001). The same study revealed a significant increase in GSH (*p* < 0.001) and the activity of GPx (*p* < 0.001), GR (*p* < 0.001), SOD (*p* < 0.001), CAT (*p* < 0.001), and PON1 (*p* < 0.001) in the pancreas concomitant with decreased protein oxidation and lipid peroxidation. Some of the findings were observed in mice models of diabetes. For instance, oral administration of 0.35 mmol of *pomegranate* for four months significantly increased PON1 gene expression (*p* < 0.05) and its activity in STZ-induced diabetes in mice fed an HFD [55]. A different mice strain (Kunming) with diabetes induced by STZ demonstrated a significant increase in AOC (*p* < 0.01), GSH (*p* < 0.01), and T-AOC (*p* < 0.01) when treated with a 400 mg/kg *pomegranate* dose for four weeks [22]. Interestingly, Balb/C mice with STZ-induced diabetes, when treated with *pomegranate*, revealed a significant decrease in macrophage peroxides (*p* < 0.05) concomitant with increased macrophagic GSH (*p* < 0.05) [52]. More recently, in HFD-STZ-induced diabetic C57BL/6 mice, administration of 20 mg/kg of punicalagin for eight weeks significantly decreased MDA (*p* < 0.01) and free fatty acid (FFA) levels (1147.2 ± 89.3 µmol/L compared to 1351.8 ± 91.6 µmol/L, *p* < 0.05) concomitant with increased total-superoxide dismutase (T-SOD) activity in the liver (*p* < 0.01). Surprisingly, this study showed no significant changes in serum T-SOD activity (*p* > 0.05) [34]. These findings collectively demonstrate the potential of *pomegranate* and its derivatives in reducing oxidative stress in rodent models of diabetes.

**The effect of *pomegranate* on markers of endothelial function in diabetic patients.**

Endothelial dysfunction contributes to the development of CVD. This study focused on sICAM-1 and sVCAM-1 from clinical trials. Our evidence in clinical trials showed conflicting findings on endothelial function following treatment with 250 mL of *pomegranate* oil in diabetes [65]. For instance, we observed a significant decrease in sICAM-1 levels from baseline (151 ± 17 ng/mL) to post treatment (138 ± 12 ng/mL, *p* < 0.001) and a decrease in sE-selectin levels from 19 ± 7 ng/mL to 13 ± 6 ng/mL post treatment (*p* < 0.001). However, there was no significant difference in sVCAM-1 levels: 27 ± 11 ng/mL compared to 31 ± 19 ng/mL (*p* > 0.05).

**The effect of *pomegranate* and its derivatives on markers of inflammation in diabetic patients.**

Inflammation is implicated in the development and progression of diabetes, especially T2D. Diabetes and inflammation have a complex relationship that involves several cellular and molecular pathways. Some implicated mechanisms include inflammatory pathways such as the activation of nuclear factor-kappa-beta (NF-κβ) [85]. Various inflammatory markers, such as TNF-α, IL-6, CRP, and NF-κβ, are evaluated to assess inflammation and serve as targets for potential therapies [85,86]. In the current review, evidence from clinical studies showed a significant decrease in the circulating levels of CRP (*p* < 0.05), IL-6 (*p* < 0.01), and TNF-α (*p* < 0.01) in diabetic patients on 8-week treatment with 500 mg of *pomegranate* peel extract [67]. Similar results were reported by a study conducted in Iran, where 12 weeks of 250 mL of PJ significantly decreased plasma hs-CRP (baseline: 3243 ± 2935 ng/mL compared to post treatment: 1791 ± 1657 ng/mL, *p* < 0.05) and IL-6 (10.9 ± 4.4 ng/L compared to 7.1 ± 5.6 ng/L, *p* < 0.05) [62]. Furthermore, this study showed a significant decrease in TNF-α between the baseline (37.0 ± 19.3 ng/L) and post-treatment (30.4 ± 17.5 ng/L) levels, *p* < 0.01. Consistently, PSO at a dose of 3g for eight weeks significantly reduced IL-6 (before treatment: 5.2 ± 2.2 pmol/mL compared to after treatment: 4.5 ± 1.9 pmol/mL, *p* = 0003) and TNF-α (before treatment: 9.2 ± 4.1 pmol/mL compared to after: 7.7 ± 2.4 pmol/mL, *p* = 0.028) in T2D patients. However, no significant differences were observed in the serum levels of hs-CRP (before: 1.4 ± 1.8 pg/mL compared to after: 0.9 ± 0.6 pg/mL, *p* = 0.11) [63]. Similar findings were acknowledged in a quasi-experimental design [61] that used concentrated PJ (50 g) for four weeks and observed a significant decrease in IL-6 levels from baseline (31.12 ± 3.12 pg/mL) to post treatment (23.40 ± 2.27 pg/mL, *p* = 0.04). Surprisingly, no statistically significant differences were observed in the levels of TNF-α (baseline: 18.72 ± 0.95 pg/mL compared to post treatment: 17.66 ± 1.41 pg/mL, *p* = 0.42) and CRP (2.37 ± 0.24 ng/ML compared to 2.44 ± 0.23 ng/ML, *p* = 0.74). Likewise, administering 250 mL of PJ for 12 weeks in diabetic patients showed no significant effect on NF-κβ [65]. Although these findings revealed some contradictory reports, the gathered evidence suggests that *pomegranate* and its derivatives may have the potential to modulate inflammation by reducing markers associated with inflammation in diabetic patients.

**The effect of *pomegranate* and its derivatives on markers of oxidative stress in diabetic patients.**

Oxidative stress plays an important role in the development and progression of both T1D and T2D due to the accumulation of ROS [5]. The accumulation of ROS results in cell, tissue, and organ damage, thus predisposing them to oxidative stress [5]. Several treatment strategies aim to target oxidative stress to minimize associated complications. In this review, we focused on the effect of *pomegranate* on various markers of oxidative stress, including MDA, SOD, CAT, and AOC, as presented in Table 2. The overall evidence gathered from clinical studies revealed that *pomegranate* exerts an antioxidant effect in patients with diabetes (Table 2). For instance, a study by Rosenblat et al. [29] showed that 50 mL of *pomegranate* juice administered for three months significantly decreased serum oxidative stress. Consistently, this was associated with lower lipid peroxide (*p* < 0.01) and TBARS levels (*p* < 0.05) and an increase in PON1 arylesterase (*p* < 0.05) activity. Furthermore, the study reported an increase in GSH levels (*p* < 0.05), concomitant with a decrease in ox-LDL uptake (*p* < 0.05), following *pomegranate* juice treatment.

Interestingly these findings were supported by Sohrab et al. [25], who reported a significant decrease in ox-LDL, increased serum TAC, and higher arylesterase activity of PON1. Partial confirmation of these results was also observed by [28], who reported increased PON1 arylesterase activity (*p* < 0.05). Similarly, administering 500 mg of *pomegranate* for three months significantly decreased MDA levels (−0.57 ± 0.55 µmol/L compared to 0.37 ± 0.48 µmol/L, *p* < 0.001) and improved antioxidant defense by increasing total plasma GSH (761.86 ± 652.71 µmol/L compared to 202.11 ± 390.76 µmol/L, *p* < 0.001) and AOC (0.45 ± 0.62 mmol/L compared to −0.09 ± 0.28 mmol/L, *p* < 0.001) [66]. These results are supported by a quasi-experimental study, as 200 mL of PJ in T2D for six weeks significantly increased PON1 (135.02 ± 104.14 μmol/L compared to 225.18 ± 149.52 μmol/L, *p* < 0.001) and arylesterase activity (165.02 ± 56.63 μmol/L compared 246.36 ± 49.26 μmol/L, *p* < 0.001), concomitant with decreasing the levels of MDA (0.073 ± 0.046 μmol/L compared to 0.029 ± 0.021 μmol/L, *p* < 0.001) [30]. More recently, Grabez et al. [67] demonstrated that 500 mg of *pomegranate* peel extract daily for eight weeks significantly ameliorated oxidative stress by reducing TBARS (baseline: 1.60 ± 0.29 µmol/mL compared to post treatment: 0.38 ± 0.15 µmol/mL, *p* < 0.001), nitrites (NO_2_^−^) (14.04 ± 2.17 nmol/mL versus 6.95 ± 1.94 nmol/mL, *p* < 0.001), and superoxide anion radical (O_2_^−^) (5.81 ± 1.09 nmol/mL compared to 3.19 ± 1.40 nmol/mL, *p* < 0.001). Moreover, the same trial showed a significant increase in TAC within the group, indicating improved antioxidant status (baseline: 149.93 ± 69.00 µmol/L versus post treatment: 230.81 ± 84.72 µmol/L, *p* < 0.05). Consistent findings were observed when 50 mL of PJ was administered for 12 weeks, as it was associated with a significant increase in TAC (22.89 ± 6.4 U/mL compared to 27.49 ± 6.8 U/mL, *p* < 0.001) and a decrease in MDA (7.69 ± 1.9 µmol/L compared to 5.79 ± 2.1 µmol/L, *p* < 0.0001) levels [26]. In addition, 150 mL of *pomegranate* extract for six weeks in T2D significantly increased the levels of GPx (414.78 ± 71.10 U/mL compared to 746.88 ± 90.24 U/L), SOD (176.50 ± 28.72 U/mL compared to 218.13 ± 22.02 U/mL), plasma GSH (5.88 ± 3.13 µmole/L compared to 10.63 ± 2.26 µmole/L), and TAC (498.75 ± 118.61 µmole/L compared to 664.37 ± 125.62 µmole/L) [64]. A quasi-experimental report also showed that administering 50 g of concentrated PJ for six weeks could increase TAC significantly (baseline: 381.88 ± 20.54 µm/L compared to post treatment: 1501 ± 146.90 µm/L, *p* < 0.001) [61]. Finally, Rock et al. [60] reported that 50 mL/day of *pomegranate* juice significantly decreased TBARS levels (*p* < 0.05), while 5 mL/day of *pomegranate* polyphenol extract for six weeks increased thiol level (*p* < 0.05), representing the serum’s AOC.

The gathered evidence suggests the potential effects of *pomegranate* and its derivatives in reducing oxidative stress in diabetic patients. These effects include reduced lipid peroxidation, increased antioxidant enzyme activity, improved glutathione levels, and enhanced TAC. To completely understand the underlying mechanisms and identify the ideal dosage and duration of *pomegranate* supplementation for efficient therapy for the management of oxidative stress, further research is required.

## 4. Discussion

Our systematic review is composed of 33 preclinical and 13 clinical studies. Indeed, more evidence supports using *pomegranate* as an antioxidant and anti-inflammatory agent, showing the benefits of improving endothelial function. Although there was no uniform evaluation of the same markers for all these parameters, the evidence gathered in this study suggests that *pomegranate* can ameliorate oxidative stress, inflammation, and endothelial dysfunction in diabetes. However, its mechanisms of action are still not well documented. When significant focus was placed on the effect of *pomegranate* on oxidative stress in rodent models of diabetes, a few markers that were considered included MDA, SOD, CAT, TBARS, GSH, GPx, TAC/AOC, ROS, PON1, FFA, and lipid peroxidation. Due to a physiological imbalance or condition, a decline in the activity of antioxidant enzymes such as GSH, SOD, CAT, and GPx enhances the vulnerability to oxidative stress.

In our review, extensive evidence from preclinical studies suggests that *pomegranate* extracts and their derivatives are good sources of antioxidant activity. This has been revealed by multiple rodent studies. These studies demonstrated a significant increase in the activity of antioxidant enzymes such as GSH [22,24,36,38,41,44,46,47,52,53,57], GPx [20,36,47,49,53], CAT [16,24,36,37,38,42,45,46,47,48,49,56], SOD [16,20,24,34,38,39,42,45,46,47,48,49,53,56,58], PON1 [53,55], and TAC [16,22,38,39,41]. Likewise, clinical evidence in diabetic patients who were treated with *pomegranate* revealed an increase in the activity of antioxidant enzymes, including GSH [29,64,66], GPx [64], PON1 [25,28,29,30], and TAC [25,26,60,61,64,66,67]. The improved activity of antioxidant enzymes supports *pomegranate* as an antioxidant in diabetes. The overall evidence indicates that *pomegranate* treatment leads to an elevation in PON1 levels in diabetes, further supporting the potential of *pomegranate* as an anti-oxidative agent. These effects are attributed to its high anthocyanin, ellagic acid, and PU content, which help counteract excessive ROS levels [60]. Therefore, this high content of secondary active compounds promotes the beneficial impact of *pomegranate* in diabetes in relation to oxidative stress. It is noteworthy that PON1 contributes to the prevention of secondary complications in diabetes, such as atherosclerosis, by hydrolyzing ox-LDL and fatty acids in the blood [87]. While such evidence is acknowledged, a contrasting report by El-Mansi et al. [56] observed a significant decrease in the activity of an antioxidant, GPx, following *pomegranate* treatment in diabetic rat embryos and their mothers. However, this might be due to the status of these animal models. On the other hand, Çukurova et al. [58] showed no effect of 2.5 mL of *pomegranate* on GSH in diabetic Sprague Dawley rats.

MDA levels were reduced in several preclinical studies [16,19,20,23,34,36,37,38,39,41,42,43,45,46,47,51,56]. Interestingly, similar findings were observed in clinical studies, as shown by a decrease in MDA levels [26,30,53,66]. Another biomarker of oxidative stress that was evaluated included TBARS. This takes into account the level of LPO degradation products in cells and tissues. In fact, diabetes is associated with increased TBARS, thus indicating increased LPO and further oxidative stress [88]. Therefore, it is imperative that LPO be inhibited in diabetes in order to alleviate oxidative stress. In the current review, TBARS levels were decreased in preclinical and clinical studies following *pomegranate* treatment in diabetes [24,29,47,48,60,67]. This shows the potential *of pomegranate* as an anti-oxidative agent in the state of diabetes.

**Figure 4:**
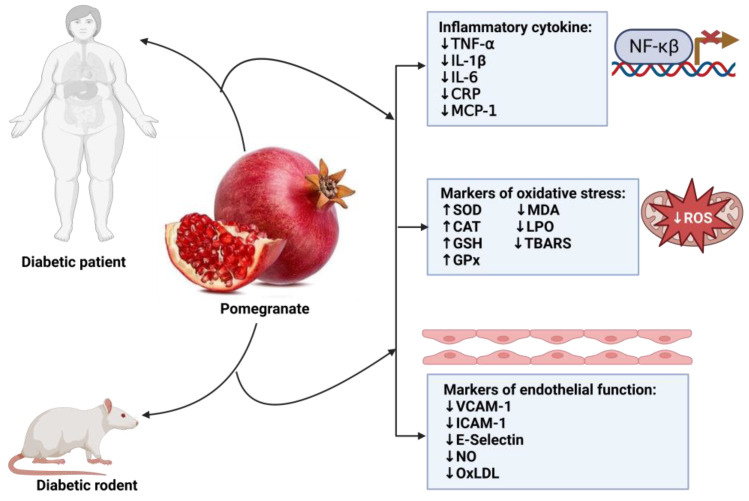
Overview of the impact of *pomegranate* on inflammation, oxidative stress, and endothelial function in diabetes. TNF-α: tumor necrosis factor-alpha; IL-1β: interleukin-1 beta; IL-6: interleukin-6; CRP: C-reactive protein; MCP-1: monocyte chemoattractant protein-1; SOD: superoxide dismutase; CAT: catalase; GSH: glutathione; GPx: glutathione peroxide; MDA: malondialdehyde; LPO: lipid peroxidation; TBARS: thiobarbituric acid reactive substances; VCAM-1 vascular cell adhesion molecule-1; ICAM-1: intercellular adhesion molecule-1; NO: nitric oxide; oxLDL: oxidized low-density lipoprotein.

Moreover, *pomegranate* treatment has been shown to alleviate oxidative stress by neutralizing ROS [21] and preventing LPO [43,53]. This beneficial effect can be explained, at least in part, by the high content of anthocyanins and ellagic acid in *pomegranate* (Figure 4). Likewise, *pomegranate* inhibits LPO, thus reducing MDA production and mitigating oxidative damage [21].

All of these findings support the use of *pomegranate* as an anti-oxidative agent, as evidenced by its ameliorative effect on the various markers of oxidative stress. The antioxidant compounds in *pomegranate* include anthocyanins and ellagic acid [89,90,91], which ameliorate oxidative stress in diabetes. A few studies suggest that anthocyanins, ellagic acid, and PU mediate *pomegranate’s* anti-oxidative stress effects by degrading and scavenging free radicals [42,92,93,94,95,96].

We found that in rodent models of diabetes, various markers of inflammation were assessed. After treatment with *pomegranate*, these markers were remarkably decreased. Some of these markers included TNF-α [35,41,42,54], IL-6 [41,42,56], IL-1β [35,42,43,54,56], NF-κβ [44,54], MCP-1 [35], and CRP [53]. Similarly, in clinical studies, we found that the administration of *pomegranate* decreased TNF-α [62,63,67], IL-6 [61,62,63,67], and CRP [62,67]. Although the results from preclinical have been replicated in clinical trials, there are still some inconsistencies in inflammation, as reported by other researchers. For instance, no significant effect of *pomegranate* was observed on TNF-α [61,62], CRP [61], hsCRP [63], and NF-κβ [65] in clinical studies. This points out the limitation of this fruit as an anti-inflammatory agent; however, some reasons may be due to the preparation of the plant extract, part of fruit used, dosage, and the stage of diabetes. Some of the modes of action of *pomegranate* in the amelioration of inflammation are mediated by anthocyanins, which inhibit the cyclooxygenase (COX), NF-κβ activity, and phosphorylation of mitogen-activated protein kinase (MAPK) proteins while inducing nitric oxide (NO) expression [97,98,99]. Regarding endothelial function, a few preclinical studies have shown reduced levels of the following endothelial markers: VCAM-1 [53], ICAM-1 [53], E-selectin [53], ET-1 [56], eNOS [58], and NO bioavailability [36,39,41]. Similarly, sICAM-1 and sE-selectin were also reduced without any effect on sVCAM-1 [65]. Reduced markers of endothelial function following *pomegranate* treatment in diabetes support its use as an agent to improve endothelial function in diabetes. One mechanism by which *pomegranates* enhance endothelial function is by reducing oxLDL levels [25].

This is partly because the accumulation of ox-LDL is associated with endothelial dysfunction and the development of atherosclerosis [100]. Therefore, our results show that *pomegranate* may preserve healthy endothelial function by preventing LDL oxidation. In clinical studies, there have been contradictory findings on oxLDL following *pomegranate* treatment. On the other hand, in a study, *pomegranate* showed no effect on eitheriNOS and eNOS [19]. For example, a report by Rosenblat [29] indicated an increase in oxLDL, whereas Sohrab [25] reported a decrease in oxLDL. Oxidized LDL impairs endothelial function and vascular health, forming foam cells and atherosclerotic plaques. This in turn may lead individuals to develop secondary complications. Due to these controversies, further research is required to better understand the effects of *pomegranate* treatment in diabetes on endothelial functions.

Although the current review has shown some of the potential benefits of *pomegranate* in diabetes, it is important to consider some of the following limitations. Firstly, most of the included studies were conducted in Asia, which comprises countries known to be major *pomegranate*-producing countries. Secondly, the diabetic rodent models presented in this study were induced through the administration of STZ or alloxan, causing T1D, unlike T2D observed in humans (as shown in Table 2). As a result, the physiological pathways leading to oxidative stress, endothelial function, and inflammation may differ between the two conditions, affecting how *pomegranate* regulates them. Additionally, the method of administration of *pomegranate*, especially in rodents, varied from different studies, with some using powder in their diet, such as *pomegranate* juice, while others prepared extracts from fruit peel and seeds. These differences in administration could contribute to conflicting findings across the studies.

Moreover, the human studies also employed different methods and doses, which led to contradicting results. For instance, some studies used *pomegranate* juice, while others used powdered *pomegranate* at varying doses. These variations in the approaches used further complicate the interpretation of the results. Amongst the strengths of the current review is the inclusion of evidence from preclinical studies and clinical trials. Evaluation of various treatment regimens also allows us to find a possible effective and safe dose. The stringent eligibility criteria, selection, and multiple database searches strengthened our review.

## 5. Conclusions

Based on a thorough examination of the various studies in this review, a comprehensive body of research consisting of 33 preclinical and 13 clinical studies involving 468 patients with T2D suggests that *pomegranate* shows promising results as a potential agent for improving oxidative stress, inflammation, and endothelial dysfunction in diabetes. Although these benefits have been acknowledged in both preclinical and clinical studies, there is limited evidence regarding endothelial function, particularly in clinical studies, which necessitates further investigation, particularly in diabetes. Additionally, it is worth noting that the clinical evidence presented in this study was based on small sample sizes ranging from 10 to 60 T2D patients, indicating that these trials may have been insufficiently powered. Therefore, based on this observation, we recommend conducting future clinical trials with larger sample sizes to gain a better understanding of the underlying mechanisms through which *pomegranate* exerts its effects and to determine the optimal dosage and duration of *pomegranate* treatment that can be used to achieve optimal anti-inflammatory and anti-oxidative benefits.

## Figures and Tables

**Table 1:** General overview of the effect of *pomegranate* extract and its active compounds on oxidative stress in rodent models of diabetes.

Author, Country	Experimental Model	Intervention and Duration	Main Findings
Rozenberg et al. [52] Israel	Streptozotocin (STZ)-induced diabetes in male Balb/C mice.	Diabetic rats were treated with *pomegranate* juice (PJ) as drinking water as a sugar fraction for ten days.	PJ sugars treatment significantly decreased macrophage peroxides and increased macrophagic glutathione (GSH).
McFarlin et al. [59] USA	Wild-type CD-1 male mice fed a high-fat diet (HFD).	HFD mice were treated with 61.79 mg of *pomegranate* for 14 weeks.	Treatment with *pomegranate* oil resulted in no significant difference in C-reactive protein (CRP).
Bagri et al. [47] India	STZ-induced diabetes in male albino Wistar rats.	500 g of powdered *pomegranate* (250 mg/kg and 500 mg/kg) were administered orally in an aqueous solution (3% *v*/*v*, 80 in water) for 21 days.	The treatment of diabetic rats with aqueous *pomegranate* (250 and 500 mg/kg) markedly decreased pancreatic thiobarbituric acid reactive substances (TBARS) and malondialdehyde (MDA) and increased glutathione peroxide (GPx), GSH, catalases (CAT), and superoxide dismutase (SOD) activity.
Mohan et al. [24] India	STZ-induced diabetes in male albino Wistar rats.	1 kilogram of *pomegranates*: the seeds were grounded to obtain juice. The concentration of the extracts was dissolved in distilled water. Diabetic rats were treated with PJ at (100 or 300 mg/kg) for four weeks.	Treatments significantly increased GSH, CAT, and SOD and decreased TBARS.
Betanzos-Cabrera et al. [55] Mexico	STZ-induced diabetes in mice fed with a high-fat diet (HFD).	Fresh PJ was prepared by diluting 12.5 mL/L in 1 L of water to make 0.35 mmol. The diluted PJ was given to the mice orally in their drinking water for four months.	Treatments significantly increased paraoxonase-1 (PON1) gene expression and its activity.
Cambay et al. [57] Turkey	STZ-induced diabetes in albino Wistar rats.	Ground powder of *pomegranate* flowers (PGFs) and powdered rat feed were mixed to make rat pellet feed. Diabetic rats were fed the mixed pellet as PGFs at 300, 400, and 500 mg/kg for eight weeks.	PGF treatment significantly decreased lipid peroxidation (LPO) and increased GSH levels.
Çukurova et al. [58] Turkey	STZ-induced diabetes in Sprague Dawley rats.	*Pomegranates* were washed, crushed, and squeezed to make PJ. Concentrated PJ was diluted in water (20 mL of concentrated juice in 500 mL of distilled water) to make 2.5 mL diluted PJ. Diabetic rats were treated with PJ for ten weeks.	PJ treatment significantly decreased endothelial nitric oxide synthase (eNOS) expression and increased SOD without significant changes in GSH.
Osman et al. [40] Egypt	Alloxan-induced diabetes in female albino rats.	*Pomegranate* peel dried and ground to powder (250 mg/kg). Seeds were used to make fresh juice (5 mL/kg). Diabetic rats were treated orally fed 250 PP mg/kg PP mixed with diet and oral PJ at 5 mL/kg) daily for four weeks.	Treatment with both regimens significantly increased total antioxidant capacity (TAC).
Shaker et al. [44] Egypt	STZ-induced diabetes in male Wistar rats.	*Pomegranate* seed extract (PSE), grounded to powder and dissolved in distilled water to form PSE (300 mg/kg/day), was given orally by gavage for four weeks.	PSE significantly decreased the pancreatic expression of nuclear factor kappa-beta (NF-κβ) and increased pancreatic GSH content.
Aboonabi et al. [16] Malaysia	STZ-nicotinamide (NAD)-induced diabetes in male Sprague Dawley rats.	The red *pomegranate* fruit was washed and peeled, and the arils were crushed and squeezed to make juice (1 mL of juice). The *pomegranate* seeds (PSs) were freeze-dried and ground into powder. The powder was dissolved into distilled water (100 mg of PSs + 1 mL DW). *Pomegranate* juice–seed (1 mL of PJ + 100 mg of PS) Diabetic rats were treated orally with *pomegranate* seeds and juice for 21 days.	Treatment with *pomegranate* significantly increased the enzymatic antioxidants, including CAT, SOD, and TAC, and decreased MDA in the plasma.
Patel et al. [45] India	STZ-induced diabetes in Wistar rat.	The leaf powder (100 g) was dissolved into methanol: water (70:30) for 72 h to obtain the hydroalcoholic extract. Diabetic rats were treated via oral route using an oral feeding needle once with 50, 100, and 200 mg/kg of ethyl acetate fraction *of Punica granatum* Linn. Leaves (EAPG) for 28 days.	EAPG significantly decreased MDA, pronounced at 200 mg/kg, while GSH, CAT, and SOD increased.
Praseytastuti et al. [51] Indonesia	STZ-induced diabetes in Sprague Dawley rats	Diabetic rats were treated orally with 1, 2, and 4 mL/200 g of PJ for four weeks.	Treatment with 2 mL/200 g of PJ significantly decreased MDA.
Ankita et al. [46] India	STZ-induced diabetes Wistar rats.	Leaf powder (100 g) was dissolved in methanol: water (70:30) for 72 h to obtain a hydroalcoholic extract. Diabetic rats were treated with a flavonoid-rich fraction of *pomegranate* leaves (PGFF) at 50, 100, and 200 mg/kg for 28 days.	PGFF significantly decreased MDA, pronounced at 200 mg/kg, while GSH, CAT, and increased SOD.
El-Missiry et al. [42] Egypt	STZ-induced diabetes in male Wistar rats.	Punicalagin (PU) powder dissolved in 0.2 mL saline solution and was intraperitoneally administered at 1 mg/kg daily for 15 days.	PU treatment significantly decreased tumor necrosis factor-alpha (TNF-α), interleukin-6 (IL-6), and MDA while increasing interleukin-1-beta (IL-1β), GSH, CAT, and SOD.
Salwe et al. [48] India	STZ-induced diabetes in male Wistar rats.	40 g of dried powder dissolved in 95% ethanol to make a hydroalcoholic extract. Leaf extract 100 and 200 mg/kg of *pomegranate*, fruit peel extract 100 mg/kg, and peel extract 200 mg/kg of *pomegranate*.	Treatment significantly increased SOD and CAT while decreasing TBARS.
Saad et al. [39] Egypt	STZ-induced insulin-dependent diabetes mellitus (IDDM) in male Swiss Albino rats.	Dried *pomegranate* peels were grounded into a fine powder, dissolved in distilled water (100 mg/1 mL), and given orally through the stomach tube to rats at a 200 mg/kg dose for 20 days.	*Pomegranate* peel powder significantly increased SOD and TAC, while MDA and nitric oxide (NO) decreased.
Wang et al. [22] China	STZ-induced diabetes in male Kunming mice.	Diabetic rats were treated with 400 mg/kg of PPE via oral gavage for four weeks.	Treatment significantly increased anti-oxidative activity, GSH, and TAC.
Mollazadeh et al. [23] Iran	STZ-induced diabetes in male Wistar rats.	Diabetic rats were orally treated daily with *pomegranate* seed oil (PSO) at 0.4 and 0.8 mg/kg for 28 days.	PSO at both concentrations significantly increased total thiol content and decreased MDA levels in the heart and kidneys.
Onal et al. [19] Turkey	STZ-induced diabetes in male Sprague Dawley rats.	Diabetic rats were treated with PJ at 100 mg kg for ten weeks.	Treatment with PJ significantly decreased MDA levels without significant changes in inducible nitric oxide synthase (iNOS) and endothelial nitric oxide synthase (eNOS) protein.
Mollazadeh et al. [49] Iran	STZ-induced diabetes in male Wistar rats.	PSO dissolved in dimethyl sulfoxide, and the rats were treated orally with PSO at 0.4 and 0.8 mL/kg for three weeks.	PSO treatment significantly increased CAT, SOD, and GPx activity and decreased oxidative stress index values in tissue and mitochondrial fractions.
Rouhi et al. [54] Malaysia	STZ-NAD-induced diabetes in male Sprague Dawley rats.	The rats were orally treated with 1 mL of PJ or 100 mg of *pomegranate* seed powder (PS) in 1 mL distilled water for 21 days.	Treatment significantly ameliorated inflammation by decreasing inflammatory markers such as TNF-α, NF-κβ, and IL-6.
Gabr et al. [37] Egypt	Alloxan-induced diabetes in male albino rats.	PJ or peel extract at 500 mg/kg orally for four weeks.	Treatment with either juice or seed extract increased CAT and decreased MDA levels.
Tugcu et al. [21] Turkey	STZ-induced diabetes in Sprague Dawley rats.	PJ of 20 mL concentrated juice in 500 mL of distilled water to make 100 μL. PJ treatment was administered at 100 μL through gastric gavage for ten weeks.	Treatment significantly increased GSH and GPx and decreased MDA without changes in SOD.
Gharib et al. [50] Iran	Alloxan monohydrate induced diabetes in Wistar rats.	*Pomegranate* fruit aqueous extract (PGE) orally with PGE for 21 days. Treated with 100, 200, and 350 mg/kg of PGE.	Treatment significantly reduced ROS generation.
El-Beih et al. [36] Egypt	STZ-NAD-induced diabetes in male Wistar albino rats.	Diabetic rats received *pomegranate* aril juice (PAJ) daily at 100 or 300 mg of PAJ/kg orally for six weeks.	PAJ at both concentrations significantly decreased MDA and NADPH oxidase (NOx) levels and increased GPx, GSH, SOD, and CAT.
El-Mans et al. [56] Saudi Arabia	STZ-induced diabetes in female Sprague Dawley rats.	50 g of *pomegranate* powder dissolved in 500 mL of methanol. Diabetic rats received 150 mg/kg/daily by gavage for 18 days.	IL-1β, IL-6, and endothelin-1 (ET-1) significantly decreased compared to the untreated diabetic group. *Pomegranate* in diabetic mothers and embryos significantly decreased the actvity of MDA and GPx and increased that of CAT and SOD.
Jin et al. [35] China	HFD-STZ-induced diabetes in C57BL/6J mice.	Punicalagin at 100, 150, and 200 mg/kg doses were administered daily through oral gavage for four weeks.	Treatment significantly decreased IL-6, TNF-α, and MCP-1 mRNA expression.
El-Deeb et al. [41] Egypt	STZ-NAD-induced diabetes in male albino Sprague Dawley rats.	850 g of *pomegranate* powder dissolved in 95% ethanol (10 × 1 L). Diabetic rats were treated orally with 200 mg/ kg of ethanolic extract for four weeks.	TNF-α, IL-6, and MDA decreased; NO, TAC, and GSH increased.
Abdulhadi et al. [53] Iraq	STZ-induced diabetes in male Wistar rats.	Diabetic rats received an intraperitoneal injection of PU at 1 mg/kg for 15 days.	PU significantly decreased the level of intercellular adhesion molecules-1 (ICAM-1), vascular adhesion molecule-1 (VCAM-1), E-selectin, CRP, monocyte chemoattractant protein-1 (MCP-1), protein, LPO and MDA while increasing the activities of GPx, SOD, GSH, and PON1 in serum.
Bagheri et al. [20] Iran	Alloxan-induced diabetes in male Wistar rats.	Hydroalcoholic *pomegranate* peel extract (PoPE), dissolving (PoPE powder 50% distilled water and 50% ethanol).	Treatment significantly increased GSH, GPx, and SOD and decreased CAT and MDA.
Zhang et al. [34] China	STZ-induced diabetes in HFD-fed male C57BL/6 mice.	Diabetic rats were orally gavaged with PU daily at (20 mg/kg body weight) for eight weeks.	PU treatment significantly decreased MDA and free fatty acid (FFA) levels in the serum and liver and increased the liver’s total-superoxide dismutase (T-SOD) activity without significant changes in serum T-SOD activity.
Abo-Saif et al. [43] Egypt	STZ-induced diabetes in male Wistar rats.	1 mL of the PoPE at a dose of 150 mg/kg orally for eight weeks,	Treatment significantly decreased lipid peroxidation, IL-1β, and MDA in the heart tissue.
Mosaoa et al. [38] Egypt	STZ-induced diabetes in female Wister rats.	Seeds (100 g) were ground in a mixer. Then, the juice was added to 1000 mL of 80% n-hexane.Diabetic rats were treated orally with three *pomegranate* hexane extracts (PHE) (25, 50, 75 mg/kg) for eight weeks.	PHE at these concentrations significantly decreased TAC and MDA and increased GSH, CAT, and SOD.

STZ: streptozotocin; PJ: *pomegranate* juice; GSH: glutathione; GPx: glutathione peroxidase; SOD: superoxide dismutase; MDA: malondialdehydes; TAC: total antioxidant capacity; ROS: reactive oxygen species, PHE: *pomegranate* hexane extract; PGF: *pomegranate* flowers; PoPE: *pomegranate* peel extract; FFA: free fatty acid; CAT: catalase: IL-1β: interleukin-1 beta; ICAM-1: intercellular adhesion molecules-1; VCAM-1: vascular adhesion molecule-1; CRP: C-reactive protein; MCP-1: monocyte chemoattractant protein-1; ET-1: endothelin; NOx: NADPH oxidase; PGE: *Pomegranate* fruit aqueous extract; iNOS: inducible nitric oxide synthase; eNOS: endothelial nitric oxide synthase; LPO: lipid peroxidation; PON1: paraoxonase-1; TBARS: thiobarbituric acid reactive substances, NF-κβ: nuclear factor kappa-beta.

**Table 2:** Overview of the effect of *pomegranate* extract and its active compounds on oxidative stress and inflammation in diabetic patients.

Author, Country	Study Design	Population	Age (Years)	BMI (kg/m^2^)	Male, n (%)	Intervention and Duration	General Findings
Rosenblat et al. [29] Israel	Case study	Ten non-insulin-dependent diabetes mellitus and ten healthy participants	50 ± 10	N/A	10 (50)	Concentrated *pomegranate* juice (PJ) was diluted (1:5) with water to make 50 mL and administered for three months.	PJ treatment significantly decreased serum oxidative stress, thiobarbituric acid reactive substance (TBARS) serum level, and oxidized lipoprotein (ox-LDL) uptake and increased glutathione (GSH) activity.
Rock et al. [60] Israel	Randomized controlled trial (RCT)	Thirty patients with type 2 diabetes (T2D)	*Pomegranate* polyphenol extract (PPE): 54 ± 3; PJ: 59 ± 2	PPE: 33 ± 2 PJ: 30 ± 3	20 (67)	PJ (50 mL) was administered daily for four weeks. PPE (5 mL) was administered daily for six weeks.	PJ consumption significantly decreased TBARS and increased thiol levels, representing the serum’s antioxidant capacity (AOC).
Sohrab et al. [65] Iran	RCT	44 T2D patients	55 ± 6.7	29.3 ± 3.9	11 (50)	250 mL of PJ daily for 12 weeks.	PJ significantly reduced soluble intercellular adhesion molecule-1 (sICAM-1) and E-selectin without any effect on soluble vascular cell adhesion molecule-1 (sVCAM-1) and nuclear factor kappa-beta (NF-κβ).
Fenercioglu et al. [66] Turkey	RCT	56 T2D and 58 healthy controls	53.51 ± 6.82	31.37 ± 4.98	22 (39)	One capsule (500 mg) containing *pomegranate* extract was administered for three months.	Treatment significantly decreased malondialdehyde (MDA) and increased antioxidant defense, total plasma glutathione (GSH), and AOC.
Fuhrman et al. [28] Israel	Quasi-experimental study	6 T2D patients	59 ± 2	30 ± 3	6 (100)	50 mL of concentrated PJ was administered daily for four weeks.	Treatment with PJ and its derivatives increased PON1 arylesterase activity.
Parsaeyan et al. [30] Iran	Quasi-experimental study	50 T2D patients	48 ± 8	30 ± 3	Not reported	200 mL of PJ was administered daily for six weeks.	PON1 and arylesterase activity significantly increased, while decreasing MDA levels.
Sohrab et al. [62] Iran	RCT	44 T2D patients	55 ± 6.7	29.4 ± 3.9	11 (50)	250 mL of PJ administered daily for 12 weeks	C-reactive protein (CRP), tumor necrosis factor-alpha (TNF-α), and interleukin-6 (IL-6) significantly decreased from baseline compared to post exposure.
Sohrab et al. [26] Iran	RCT	22 T2D, 22 healthy controls	59 ± 6.7	29.49 ± 3.9	11 (50)	250 mL of PJ was administered daily for 12 weeks.	PJ significantly increased total antioxidant capacity (TAC) and decreased MDA.
Shishehbor et al. [61] Iran	Quasi-experimental	31 T2D patients	46 ± 8.3	29.53 ± 0.69	15 (48)	50 g of concentrated *pomegranate* juice administered daily for four weeks	PJ significantly increased TAC and decreased IL-6, while TNF-α and CRP were not different between baseline and post treatment.
Sohrab et al. [25] Iran	RCT	30 T2D and 30 healthy controls	54.6 ± 8.4	27.2 ± 3.4	15 (50)	200 mL of PJ was administered daily for six weeks.	PJ treatment significantly decreased ox-LDL and anti-ox-LDL antibodies and TAC, while the PON-1 activity increased significantly.
Yarmohamadi et al. [64] Iran	Semi-experimental study	33 T2D patients	56.50 ± 3.85	26.42 ± 4.79	0 (0)	150 mL of *pomegranate* extract was administered daily for six weeks.	*Pomegranate* extract treatment significantly increased glutathione peroxidase (GPx), SOD, plasma GSH, and TAC levels.
Khajebishak et al. [63] Iran	RCT	52 obese T2D patients	44.6 ± 5.1	33.96 ± 4.9	9 (34.6)	3 g *pomegranate* seed oil (PSO) was administered daily for eight weeks.	PSO treatment significantly decreased IL-6 and TNF-α with no significant changes in high-sensitivity C-reactive protein (hs-CRP).
Grabez et al. [67] Bosnia & Herzegovina	RCT	60 T2D patients	57.87 ± 6.08	30.95 ± 4.37	15 (50)	*Pomegranate* peel extract (PoPEx) containing a capsule (250 mg) was administered twice daily for eight weeks.	Treatment significantly decreased TBARS, nitrites (NO_2_^−^), superoxide anion radical (O_2_^−^), CRP, IL-6, and TNF-α while increasing TAC.

PJ: *pomegranate* juice; GSH: glutathione; GPx: glutathione peroxidase; SOD: superoxide dismutase; MDA: malondialdehydes; TAC: total antioxidant capacity; AOC: antioxidant capacity; RCT: randomized controlled trial; BMI: body mass index; T2D: type 2 diabetes mellitus; ROS: reactive oxygen species, PSO: *pomegranate* seed oil; CAT: catalase: IL-1β: interleukin-1; CRP: C-reactive protein; hs-CRP: high-sensitivity C-reactive protein; NO_2_^−^: Nitrates; PoPEx: *pomegranate* peel extract; PPE: *pomegranate* polyphenol extract; LPO: lipid peroxidation; PON1: paraoxonase-1; TBARS: thiobarbituric acid reactive substances, oxLDL: oxidized low-density lipoprotein; WPOMx1: Wonderful variety of *pomegranate* polyphenol extract; N/A: not applicable; sICAM-1: soluble intercellular adhesion molecule-1; sVCAM-1: soluble vascular cell adhesion molecule-1; NF-κβ: nuclear factor kappa-beta.

## Supplementary Materials

The following supporting information can be downloaded at: https://www.mdpi.com/article/10.3390/antiox12081566/s1. Table S1: Search on Scopus for oxidative stress; Table S2: Search on Scopus for endothelial function; Table S3: Search on Scopus for inflammation; Table S4: Search on PubMed for oxidative stress; Table S5: Seach on PubMed for inflammation; Table S6: Search on PubMed for endothelial function.

## Abbreviations

DM: diabetes mellitus; T2D: type 2 diabetes; STZ: streptozotocin; NAD: nicotinamide; TNF-α: tumor necrosis factor-alpha; IL-1β: interleukin-1 beta; IL-6: interleukin-6; CVD: cardiovascular disease; ROS: reactive oxygen species; PON1: paraoxonase-1; FFA: free fatty acid; LDL: low density lipoprotein; CRP: C-reactive protein; MCP-1: monocyte chemoattractant protein-1; SOD: superoxide dismutase; CAT: catalase; GSH: glutathione; GPx: glutathione peroxide; MDA: malondialdehyde; LPO: lipid peroxidation; TBARS: thiobarbituric acid reactive substances; VCAM-1: vascular cell adhesion molecule-1; sVCAM-1: soluble vascular cell adhesion molecules-1; ICAM-1: intercellular adhesion molecule-1; sICAM-1: soluble intercellular adhesion molecule-1; NOx: NADPH oxidase; NO: nitric oxide; oxLDL: oxidized low-density lipoprotein; COX: cyclooxygenase; AGEs: advanced glycation end product; IKK: inhibitor of nuclear factor-κ; NF-κβ: nuclear factor kappa β; ROS: reactive oxygen species; oxLDL: oxidized lipoprotein; PKA: protein kinase; PSE: *pomegranate* peel extract; TAC: total antioxidant capacity; PJ: *pomegranate* juice; MAPK: mitogen-activated protein kinase; ET-1: endothelin-1; eNOS: endothelial nitric oxide synthase; iNOS: inducible nitric oxide synthase; PoPEx: *pomegranate* peel extract; PSO: *pomegranate* seed oil; NO_2_^−^: nitrites; WPOMx1: Wonderful variety of *pomegranate* polyphenol extract; PPE: *pomegranate* peel extract; PHE: *pomegranate* hexane extracts; PAJ: *pomegranate* aril juice; PU: punicalagin; IDDM: insulin-dependent diabetes mellitus; SGLT2: sodium-glucose cotransporter-2; WHO: world health organization; TCAM: traditional, complementary, and alternative medicines; IDF: International Diabetes Federation.
