# Supplementary material for: The Impact of Punica granatum Linn and Its Derivatives on Oxidative Stress, Inflammation, and Endothelial Function in Diabetes Mellitus: Evidence from Preclinical and Clinical Studies
Source: Antioxidants (Basel). 2023 Aug 4;12(8):1566. doi: 10.3390/antiox12081566 (PMC10451900; doi:10.3390/antiox12081566)
Supplement: Supplementary file 1 [file antioxidants-12-01566-s001.zip › antioxidants-2465421-supplementary.pdf]

Review

The Impact of *Punica granatum Linn* and its Derivatives on Oxidative Stress, Inflammation, and Endothelial Function in Diabetes Mellitus: Evidence from Preclinical and Clinical Studies

Table S1. Search on Scopus for oxidative stress

**Terms**	**Search terms**	**Search with limitation**	**Records**
1	*pomegranate*	TITLE-ABS-KEY(pomegranate) AND TITLE-ABS-KEY(oxidative stress) AND TITLE-ABS-KEY(diabetes mellitus)) AND (LIMIT-TO (DOCTYPE,"ar")) AND (LIMIT-TO (LANGUAGE,"English")) AND (LIMIT-TO (SRCTYPE,"j"))	50
2	Oxidative stress		
3	Diabetes mellitus		

Table S2. Search on Scopus for endothelial function

Terms	Search terms	Search with limitation	Records
1	*pomegranate*	(TITLE-ABS-KEY (pomegranate) AND TITLE-ABS-KEY (endothelial AND function) AND TITLE-ABS-KEY (diabetes AND mellitus)) AND (LIMIT-TO (DOCTYPE , "ar")) AND (LIMIT-TO (SRCTYPE , "j"))	2
2	Endothelial function		
3	Diabetes mellitus		

Table S3. Search on Scopus for inflammation

**Terms**	**Search terms**	**Search with limitation**	**Records**
1	*pomegranate*	(TITLE-ABS-KEY (pomegranate) AND TITLE-ABS-KEY (inflammation) AND TITLE-ABS-KEY (diabetes AND mellitus)) AND (LIMIT-TO (DOCTYPE , "ar")) AND (LIMIT-TO (SRCTYPE , "j"))	43
2	Inflammation		
3	Diabetes mellitus		

Table S4. Search on PubMed for oxidative stress

**Terms**	**Search terms**	**Search with limitation**	**Records**
1	*pomegranate*	(("pomegranate"[MeSH Terms] OR "pomegranate"[All Fields] OR "pomegranates"[All Fields]) AND ("oxidative stress"[MeSH Terms] OR ("oxidative"[All Fields] AND "stress"[All Fields]) OR "oxidative stress"[All Fields]) AND ("diabetes mellitus"[MeSH Terms] OR ("diabetes"[All Fields] AND "mellitus"[All Fields]) OR "diabetes	34
2	Oxidative stress		
3	Diabetes mellitus		

		mellitus"[All Fields])) AND ((1000/1/1:2023/5/20[pdat]) AND (english[Filter]))	

Table S5. Seach on PubMed for inflammation

Terms	Search terms	Search with limitation	Records
1	*pomegranate*	("pomegranate"[MeSH Terms] OR "pomegranate"[All Fields] OR "pomegranates"[All Fields]) AND ("inflammation"[MeSH Terms] OR "inflammation"[All Fields] OR "inflammations"[All Fields] OR "inflammation s"[All Fields]) AND ("diabetes mellitus"[MeSH Terms] OR ("diabetes"[All Fields] AND "mellitus"[All Fields]) OR "diabetes mellitus"[All Fields])	21
2	Inflammation		
3	Diabetes mellitus		

Table S6. Search on PubMed for endothelial function

Terms	Search terms	Search with limitation	Records
1	*pomegranate*	("pomegranate"[MeSH Terms] OR "pomegranate"[All Fields] OR "pomegranates"[All Fields]) AND (("endothelialization"[All Fields] OR "endothelialize"[All Fields] OR "endothelialized"[All Fields] OR "endothelializing"[All Fields] OR "endothelials"[All Fields] OR "endothelium"[MeSH Terms] OR "endothelium"[All Fields] OR "endothelial"[All Fields]) AND ("functional"[All Fields] OR "functional s"[All Fields] OR "functionalities"[All Fields] OR "functionality"[All Fields] OR "functionalization"[All Fields] OR "functionalizations"[All Fields] OR "functionalize"[All Fields] OR "functionalized"[All Fields] OR "functionalizes"[All Fields] OR "functionalizing"[All Fields] OR "functionally"[All Fields] OR "functionals"[All Fields] OR "functioned"[All Fields] OR "functioning"[All Fields] OR "functionings"[All Fields] OR "functions"[All Fields] OR "physiology"[MeSH Subheading] OR "physiology"[All Fields] OR "function"[All Fields] OR "physiology"[MeSH Terms])) AND ("diabetes mellitus"[MeSH Terms] OR ("diabetes"[All Fields] AND "mellitus"[All Fields]) OR "diabetes mellitus"[All Fields])	3
2	Endothelial function		
3	Diabetes mellitus		
